# Signs of ongoing inflammation in female patients with chronic widespread pain: A multivariate, explorative, cross-sectional study of blood samples

**DOI:** 10.1097/MD.0000000000006130

**Published:** 2017-03-03

**Authors:** Björn Gerdle, Bijar Ghafouri, Nazdar Ghafouri, Emmanuel Bäckryd, Torsten Gordh

**Affiliations:** a Pain and Rehabilitation Centre, and Department of Medical and Health Sciences, Linköping University, Linköping; b Department of Surgical Sciences, Uppsala University, Uppsala, Sweden.

**Keywords:** chemokine, cytokine, fibromyalgia, pain, widespread pain

## Abstract

This cross-sectional study investigates the plasma inflammatory profile of chronic widespread pain (CWP) patients compared to healthy controls (CON). Rather than analyzing a relatively few substances at a time, we used a new multiplex proximity extension assay (PEA) panel that enabled the simultaneous analysis of 92 inflammation-related proteins, mainly cytokines and chemokines.

Seventeen women with CWP and 21 female CON participated and a venous blood sample was drawn from all subjects. Pain intensity and pain thresholds for pressure, heat, and cold were registered. A PEA panel (92 proteins) was used to analyze the blood samples. Multivariate data analysis by projection was used in the statistical analyses.

Eleven proteins significantly differentiated the CON and CWP subjects (*R*^2^ = 0.58, *Q*^2^ = 0.37, analysis of variance of cross-validated predictive residuals *P* = 0.006). It was not possible to significantly regress pain thresholds within each group (CON or CWP). Positive significant correlations existed between several proteins and pain intensities in CWP, but the model reliability of the regression was poor.

CWP was associated with systemic low-grade inflammation. Larger studies are needed to confirm the results and to investigate which alterations are condition-specific and which are common across chronic pain conditions. The presence of inflammation could promote the spreading of pain, a hallmark sign of CWP. As it has been suggested that prevalent comorbidities to pain (e.g., depression and anxiety, poor sleep, and tiredness) also are associated with inflammation, it will be important to determine whether inflammation may be a common mediator.

## Abstract 2

Supplemental Digital Content is available in the text

## 1 Introduction

According to the criteria of the American College of Rheumatology, about 2% of the general population fulfils the criteria for fibromyalgia syndrome (FMS).^[1]^ Chronic widespread pain (CWP), which includes FMS, is even more prevalent, affecting approximately 10% of the general population.^[2–5]^ The clinical picture of CWP is dominated by widespread pain in the body, and it often includes other somatic and psychological symptoms such as depression and anxiety, poor sleep, fatigue, and reduced cognitive capacity. Moreover, patients with CWP/FMS often report partial or whole work incapacity, increased sick-leave, and poor quality of life and health.^[6–8]^ Clearly, CWP is a major health burden.

The mechanisms underlying the transition from a local pain condition to CWP are largely unknown. Risk factors for CWP include increasing age, female sex, family history of pain, low physical activity, obesity, and depression.^[5,9]^ Typically, CWP (and chronic pain in general) is characterized by central hyperexcitability in the nociceptive systems.^[10–13]^ In FMS, the top-down pain modulating systems appear to be impaired,^[14]^ a condition that may contribute to central hyperexcitability. This is not to say that peripheral nociceptive input is unimportant; indeed, alterations in muscle biochemistry and peripheral nociceptors have been reported^[15–22]^ and on-going peripheral input seems necessary if central hyperexcitability and other central alterations are to be maintained.^[23]^ Indeed, both peripheral and central mechanisms may be important in the pathophysiology of CWP/FMS. In CWP research, chronic inflammation is an important area of investigation.^[24,25]^ For example, several studies have found elevated plasma and/or serum levels of the proinflammatory cytokines interleukin (IL)-6 and IL-8^[24,25]^ in patients with FMS, and one study has found elevated IL-8 in cerebrospinal fluid of patients with FMS.^[26]^

This study compares the plasma inflammatory profile of CWP patients with the plasma inflammatory profile of healthy controls (CON). Rather than analyzing relatively few substances at a time, we used a new multiplex proximity extension assay (PEA) panel that enables the simultaneous analysis of 92 inflammation-related proteins, mainly cytokines and chemokines.^[27]^ This panel has been used previously in patients with chronic radicular pain,^[28]^ showing ongoing inflammation detectable in blood in the patients but not in the pain-free controls. Within this aim, we investigated whether the analyzed proteins correlated with pain intensity and pain thresholds for pressure, cold, and heat. To analyze data from a systems biology perspective^[29]^ (i.e., analyzing all variables simultaneously, not one at a time), we used advanced multivariate data analysis by projection (MVDA).^[30–32]^

## 2 Materials and methods

This cross-sectional study is a subproject of a comprehensive project. Earlier reports from this cohort—CWP (n = 19) and CON (n = 24)—have reported results concerning metabolic and endocannbionoid-related anti-inflammatory lipids in muscle interstitium of the trapezius muscle and proteomic alterations in muscle tissue.^[16,18,19,33,34]^ That is, background data and data concerning pain intensity ratings, psychological aspects, and pressure pain thresholds have been presented earlier (although not exactly for the same number of subjects). This information is indicated when data for these variables are presented in the present manuscript.

### 2.1 Procedures

Primary screening was accomplished using a self-reported pain questionnaire and a structured telephone interview. To confirm the subjects’ eligibility, they agreed to a standard clinical examination that collected data such as weight, height, and blood pressure. All participants answered questionnaires addressing chronic pain. In addition, their upper and lower extremities were measured with respect to pain thresholds for different stimuli. Subjects then participated in a microdialysis experiment that collected biochemical data from the trapezius muscle (these data are reported elsewhere),^[16]^ and during this experiment, a blood sample was drawn. It is this blood sample that the present study analyzed.

### 2.2 Subjects

Subjects with CWP were recruited for the study either among former patients with CWP at the Pain and Rehabilitation Center of the University Hospital, Linköping, Sweden or from an organization for FMS patients. From this recruitment procedure, 19 women with CWP agreed to participate in the study; for 17 of these women, we collected adequate blood samples. Inclusion criteria were female sex, age between 20 and 65 years, and widespread pain according to the ACR classification.^[35]^ All patients were clinically examined by 1 of 2 physicians. The 18 tender points according to the FMS classification criteria of ACR 1990^[35]^ were also clinically examined for sensitivity to manual pressure. Fifteen of 17 CWP subjects also fulfilled the ACR criteria for FMS.^[35]^

The 24 CONs were recruited through advertisement in the local newspaper. For 21 of these, we collected adequate blood samples. The inclusion criteria for the control subjects were female sex, age between 20 and 65 years, and pain-free. A medical history was taken that included any current or previous presence of pain or discomfort in the neck and shoulder region.

For both the CWP and CON groups, a clinical examination was used to check inclusion and exclusion criteria. Exclusion criteria in both groups were any kind of anticoagulatory use, continuous anti-inflammatory drugs, opioid, or steroidal use, bursitis, tendonitis, capsulitis, postoperative conditions in the neck/shoulder area, previous neck trauma, disorder of the spine, neurological disease, rheumatoid arthritis or any other systemic diseases, metabolic disease, malignancy, severe psychiatric illness, pregnancy, and difficulties understanding the Swedish language.

As mentioned above, this study is a part of a comprehensive project. The ethical application, following the Swedish laws and regulations, required a sample size calculation to insure sufficient power. The number of subjects needed to achieve sufficient power was based on the concentration of interstitial lactate of the trapezius in healthy controls and in patients with chronic trapezius myalgia (data retrieved from our previous studies).^[36]^ Using the Power and Sample Size Calculation ver. 3.0.2^[37]^ and 4 parameters (alpha = 0.05, power = 0.8, difference between groups = 1.7, and SD = 1.7), we found that we needed a minimum of 17 subjects in each group. As described in statistics section, advanced multivariate analyses have been applied, but multivariate sample size programs are to the best of our knowledge not available in commercial statistical programs.

After receiving verbal and written information about the study, all subjects signed a consent form that was in accordance with the Declaration of Helsinki. The study was granted ethical clearance by the Linköping University Ethics Committee (Dnr: M10–08, M233–09, Dnr: 2010/164–32).

## 3 Methods

### 3.1 Primary screening

The Nordic Ministry Council Questionnaire (NMCQ) was used in the primary screening.^[38]^ This instrument is a well-established self-reported pain questionnaire that assesses low back, neck, shoulder, and general complaints. It includes symptoms (ache, pain, and discomfort) arising from 9 well-defined anatomical regions. The participants marked the regions where they experienced pain the previous 7 days and the previous 12 months.

### 3.2 Questionnaire on health and function

The participants completed a brief questionnaire with questions regarding anthropometric data, pain intensity, depressive and anxiety symptoms, catastrophizing, and quality of life (Table 1). The questionnaire included the Swedish validated versions of the instruments. For references concerning the different instruments including their psychometric properties, see earlier studies from our group.^[39,40]^

**Table 1:**
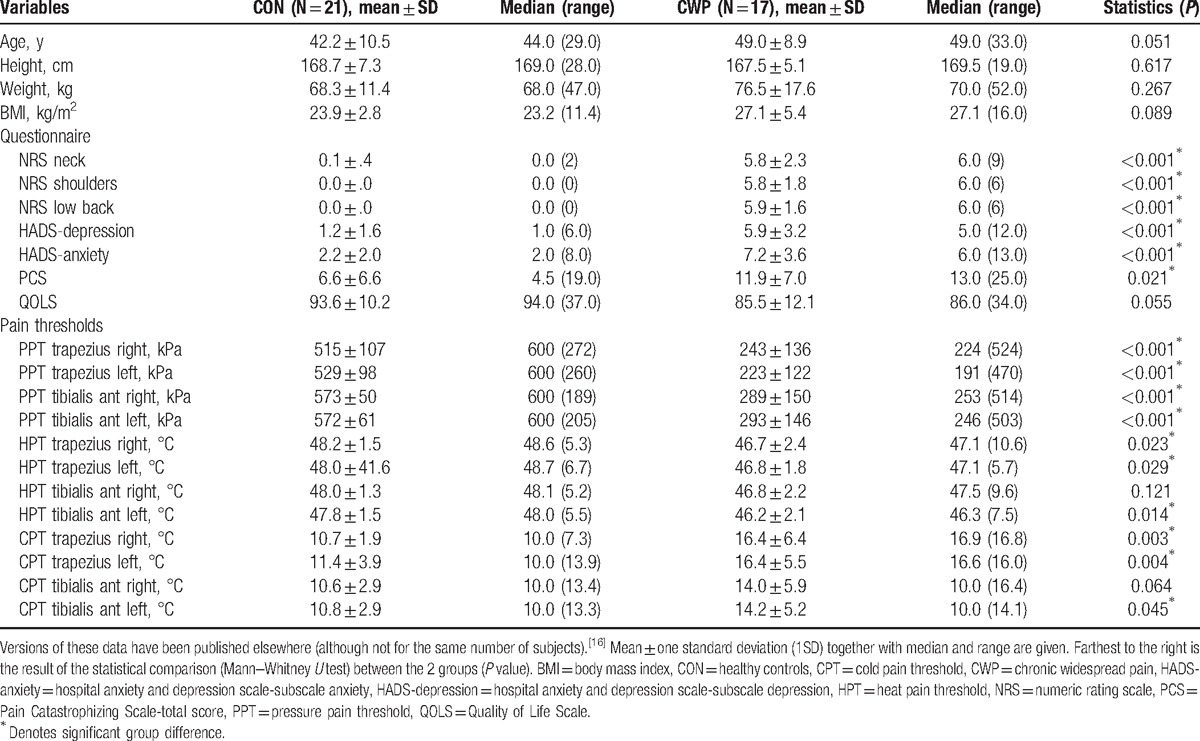
Age and anthropometric data together with pain intensities, psychological symptoms (depression, anxiety, and catastrophizing), and quality of life gathered from a questionnaire together with registrations of pain thresholds for pressure, heat, and cold for the CON and the patients with CWP.

#### 3.2.1 Pain intensity ratings

The subjects rated their neck, shoulder, and low back pain intensity the previous 7 days using a numeric rating scale (NRS) (0 = no pain and 10 = worst possible pain).

#### 3.2.2 Anxiety and depressive symptoms

The Hospital Anxiety and Depression Scale (HADS) is a short self-assessment questionnaire that measures anxiety and depression. HADS comprises 7 items in each of the depression (HAD-D) and anxiety (HAD-A) scales. Possible subscale scores range from 0 to 21, with the lower score indicating the least depression and anxiety possible. A score of ≤7 indicates a non-case, a score of 8 to 10 indicates a doubtful case, and a score of ≥11 indicates a definite case.

#### 3.2.3 Catastrophizing

The Pain Catastrophizing Scale (PCS) measures aspects of catastrophizing such as rumination, magnification, and helplessness. In the present study, the total PCS score was used; a score of 52 was the maximum. A cutoff score of 38 is generally considered to indicate catastrophizing.

#### 3.2.4 Quality of life

The Quality of Life Scale (QOLS) is composed of 16 items that together describe the quality of life concept: material comforts; health; relationships with parents, sibling, and other relatives; having and rearing children; close relationships with spouse or significant others; close friends; helping and encouraging others, participating in organizations, and volunteering; participating in political organizations or public affairs; learning; understanding yourself; work; expressing yourself creatively; socializing; reading, listening to music, or watching entertainment; participating in active recreation; and independence, being able to do things for yourself. Subjects estimated their satisfaction with their current situation. A 7-point satisfaction scale is used for each item. The item scores are added to a total score, ranging from 16 to 112. A higher total score reflects higher satisfaction.

### 3.3 Pain thresholds for pressure, cold, and heat

Algometry for determining pressure pain thresholds (PPTs) was performed using an electronic pressure algometer (Somedic, Hörby, Sweden) as previously described.^[41]^ The diameter of the contact area was 10 mm and the pressure was applied perpendicularly to the skin at a speed of 30 kPa/s. The subjects were instructed to mark the PPT by pressing a button as the sensation of “pressure” changed to “pain.” Algometry was performed bilaterally over the medial, middle, and lateral part of the descending part of the trapezius muscle and over the tibialis anterior to determine the PPTs. All PPT measurements were conducted twice in approximately 5-minute intervals. The PPT values were calculated as the mean of these 2 measurements of the lateral, middle, and medial site on the right and left trapezius muscle and of the right and left tibialis anterior muscles. In the multivariate regression analyses, the mean values of the right and left trapezius measurements were used. Before the actual PPT testing, the subjects were given instructions and allowed to examine the testing procedure.

Thermal sensory testing was performed using a modular sensory analyzer from Somedic, Hörby, Sweden. The method applied in the present study has previously been described in detail.^[42,43]^ Pain thresholds for cold (CPTs) and heat (HPTs) were determined. The testing was conducted over the upper part of the trapezius muscle (bilaterally approximately midway on a line between C7 and the acromion) and over the anterior tibialis muscle (bilaterally approximately 7–10 cm below the patella). All tests were conducted on all participants according to a structured protocol and performed according to the Marstock method.^[44]^ A thermode with a stimulating surface of 25 × 50 mm, consisting of Peltier elements and a temperature change range of 1°C/s, was used for all tests. The stimulating surface could be either warmed or cooled depending on the direction of the current flow through the semiconductor junctions; the participant could reverse the direction of the current by pressing a button per the specific instructions given. During the tests, the participants sat comfortably in a quiet room with an ambient temperature of approximately +22°C. All tests were performed in the same order by a skilled research nurse. The measurements of pain thresholds were conducted a few days before the blood samples were collected.

### 3.4 Blood samples

A description of the microdialysis (MD) session has been extensively presented elsewhere.^[16,34]^ Both the CWP and CON participants were asked not to perform any strenuous exercise 2 days before the study. They were also instructed not to drink any beverages with caffeine, not to smoke, and not to take paracetamol medication on the day of the study. In addition, they were asked not to take any NSAID-medication the week before the study. After the insertion of 2 catheters into the trapezius muscle, participants rested for 140 minutes (i.e., the trauma period + baseline). The baseline period (20 minute) was followed by a 20-minute period of standardized repetitive low-force exercise. The experiment ended with a recovery period of 60 minutes during which participants rested. A venous blood sample was drawn 200 minutes into the MD session (i.e., in the middle of the recovery period). After centrifugation, the plasma sample was stored as aliquots at −70°C until analysis.

### 3.5 Proximal extension assay

In the present study, we took advantage of the multiplex PEA technology in which 92 proteins (see Table in Supplemental Digital Content 1 which shows the panel of 92 proteins) are simultaneously analyzed.^[4,38]^ The multiplex PEA was conducted using Proseek Multiplex Inflammation I (Olink Bioscience, Uppsala, Sweden) per the manufacturer's instructions. Briefly, a 1-μL sample was mixed with a 3-μL incubation mix containing 94 probe pairs (each pair consisting of 2 target-specific antibodies equipped with unique barcoded DNA oligonucleotides). The mixture was incubated at 8°C overnight. Then, a 96-μL extension mix containing PEA enzyme and PCR reagents was added and incubated for 5 minutes at room temperature before the plate was transferred to a thermal cycler for an extension reaction followed by 17 cycles of DNA amplification. A 96.96 Dynamic Array IFC (Fluidigm, South San Francisco, CA) was prepared and primed per the manufacturer's instructions. In a new plate, a 2.8-μL sample mixture was mixed with a 7.2-μL detection mix from which 5 μL was loaded into the right side of the primed 96.96 Dynamic Array IFC. Primer pairs (5 μL), unique for each assay, were loaded into the left side of the 96.96 Dynamic Array IFC, and the protein expression program was run in Fluidigm Biomark reader per the instructions for Proseek. The Biomark run was verified Multiplex. Data are expressed as Normalized Protein eXpression (NPX). NPX values are obtained by normalizing cq-values against extension control, interplate control, and a correction factor. NPX values are on log2 scale where a high NPX value corresponds to a high-protein concentration and can be linearized using the polymerase chain reaction analyzing software (Fluidigm, CA). The data were exported to a spreadsheet. The normalized protein expression (NPX) values were used for further statistical analysis. Substances that were detected in at least 60% of the samples were included in the statistical analyses.

### 3.6 Statistical analysis

Statistical analyses were made using IBM SPSS (version 23.0; IBM Corporation, Route 100 Somers, NY) and SIMCA-P+ (version 13.0; Umetrics Inc, Umeå) and *P* ≤ 0.05 was used as level of significance in all analyses. The nonparametric Mann–Whitney *U* test was used to compare background data and clinical variables between the CWP and the CON subjects. Data are presented as mean ± one standard deviation (±1 SD) together with median and range.

Traditional univariate statistical methods can quantify level changes of individual substances, but disregard interrelationships between them and thereby ignore system-wide aspects. Moreover, traditional statistical methods (e.g., multiple and logistic regression) have obvious problems handling data sets with more variables than subjects (i.e., datasets characterized as short and broad). Therefore, we used advanced MVDA using SIMCA-P+ version 13.0 (Umetrics AB, Umeå, Sweden). When applying MVDA, we followed the recommendations concerning omics data presented by Wheelock and Wheelock.^[30]^ Variables were mean-centered and scaled for unified variance (UV-scaling). An unsupervised principal component analysis (PCA) was used to detect whether moderate or strong outliers existed among all the observations. Orthogonal partial least squares discriminant analysis (OPLS-DA) was performed to regress group membership using the proteins as regressors. Orthogonal partial least squares (OPLS) were used when regressing PPT, HPT, CPT, and NRS using the proteins as regressors. Regressors with regression coefficients with a jack-knifed 95% confidence interval not including 0 and the variable influence on projection (VIP) value exceeding 1 were considered important. The OPLS-DA analysis was made in 2 steps. First, from the analysis all the proteins we selected proteins with VIP >1.0 combined with the jack-knifed confidence intervals in the coefficients plot not including zero. Second, these proteins were used in a new regression, which is presented in the results. Coefficients (PLS scaled and centered regression coefficients) were used to note the direction of the relationship (positive or negative). The Tables also present p(corr) for each significant protein. This is the loading of each variable scaled as a correlation coefficient and thus standardizing the range from −1 to +1. P(corr) is stable during iterative variable selection and comparable between models. An absolute p(corr) >0.4 to 0.5 is generally considered significant.^[30]^*R*^2^ describes the goodness of fit—the fraction of sum of squares of all the variables explained by a principal component. *Q*^2^ describes the goodness of prediction—the fraction of the total variation of the variables that can be predicted by a principal component using cross validation methods. *R*^2^ should not be considerably higher than *Q*^2^; if *R*^2^ is substantially greater than *Q*^2^ (a difference >0.3),^[32]^ the robustness of the model is poor, implying overfitting.^[30]^ SIMCA-P+ calculates and cross-validates different components (latent variables), but these are only presented if they are significant according to specified rules.^[32]^ Moreover, analysis of variance of cross-validated predictive residuals (CV-ANOVA), which is a SIMCA-P+ diagnostic tool for assessing model reliability, was also computed. CV-ANOVA provides a familiar *P* value metric for the model.^[30]^ The presentation of the MVDA results in the tables has been complemented with a traditional nonparametric statistical test (i.e., the Mann–Whitney *U* test) for group comparisons and the Spearman rank correlation test for correlation analyses.

## 4 Results

### 4.1 Background data

No significant differences in age or anthropometric variables existed between CON and CWP (Table 1). As expected, significant differences in pain intensities between the 2 groups were found (Table 1). Although significant differences existed in the 2 subscales of HADS, the values at the group level were well below the cutoff limits generally applied for these subscales (Table 1). A significant group difference was found for PCS, but both groups had mean and median values well below the cutoff score of 38. CWP reported a significantly worse situation in quality of life (QOLS).

### 4.2 Pain thresholds

Highly significant group differences existed in PPT at the anatomical sites investigated (i.e., trapezius and tibialis anterior muscles bilaterally) (Table 1). CPT and HPT showed relatively prominent group differences in the upper part of the body whereas in the lower body the group differences were less systematic and less pronounced (Table 1).

### 4.3 Number of proteins

In the present study, we obtained valid data for 73 of 92 proteins (see table in Supplemental Digital Content 1, which shows the panel of 92 proteins).

### 4.4 Check of multivariate outliers

The PCA found no indications of multivariate outliers (data not shown).

### 4.5 Proteins important for differentiating between groups (CON or CWP)

Using 24 substances as regressors after the 2-step procedure, the OPLS-DA regression identified 11 proteins that were significant for the differentiation of the subjects into CON or CWP (Table 2) (cf. Statistics). This regression—with 2 latent variables (one predictive interclass and one orthogonal infraclass)—had a relatively high explained variation (fit) and predictivity (*R*^2^ = 0.58 and *Q*^2^ = 0.37) (Table 2) and it was highly significant according to the CV-ANOVA. Hence, it was possible to significantly predict group membership (CON or CWP) using certain proteins.

**Table 2:**
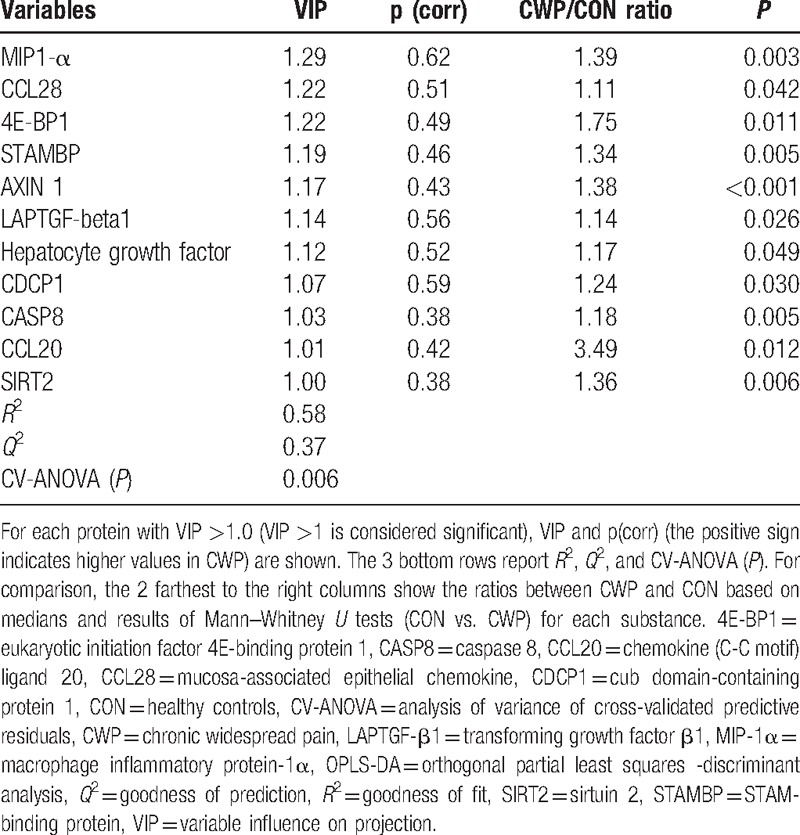
OPLS-DA regression of group membership (CON [denoted 0] or CWP [denoted 1]) using 24 proteins as regressors (*x*-variables).

Using only the 11 substances with VIP >1.0 as regressors (i.e., the substances displayed in Table 2), we found a significant OPLS-DA regression (*R*^2^ = 0.37, *Q*^2^ = 0.33; CV-ANOVA (*P*) = 0.00128; 1 predictive interclass component).

### 4.6 Proteins correlating with PPT

Since PPTs showed prominent group differences (Table 1) and it was possible to assign group membership based on PPTs, we expected a significant regression model with prominent similarities to the regression of group membership. Thus, it was possible to significantly predict the mean of the 4 PPT variables in both groups taken together (Table 3) and the identified and significant proteins were identical to the most important proteins in the regression of group membership (Table 2). The significant regression with 2 latent variables (1 predictive interclass and 1 orthogonal intraclass) explained 44% of the variation in PPT (i.e., *R*^2^ = 0.44) based on 18 proteins after the 2-step procedure. Negative correlations existed between the proteins displayed in Table 3 and PPTs; the 6 most important proteins (VIP >1) are shown in Table 3.

**Table 3:**
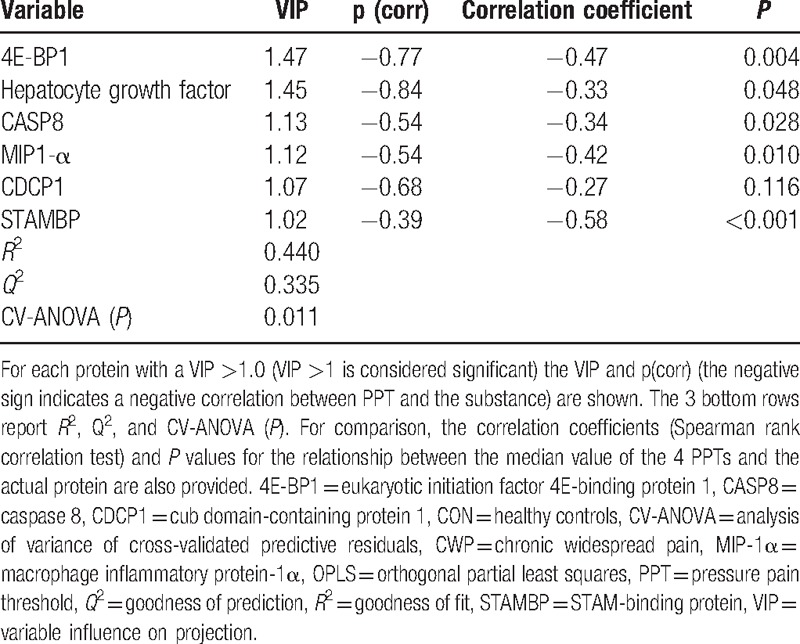
OPLS regression of the mean of 4 PPTs (trapezius bilateral and tibialis anterior bilateral) using 18 proteins as regressors (*x*-variables) in all subjects taken together.

Using only the 6 substances shown in Table 3 as regressors of the mean of PPTs, we found a significant regression (*R*^2^ = 0.38, *Q*^2^ = 0.35; CV-ANOVA (*P*) = 0.0088; 1 predictive interclass and 1 orthogonal intraclass component). It was not possible to significantly regress the mean of the 4 PPT variables within each group (CON or CWP).

### 4.7 Proteins versus CPT and HPT

No stable and significant regressions were obtained for all subjects taken together or in the 2 groups separately.

### 4.8 Proteins correlating with pain intensity (neck, shoulders, and low back)

Positive correlations existed between several proteins and pain intensities in CWP (Table 4), even though the explained variation was lower than in the regressions presented in Table 2 and Table 3. Hence, the final regression model with 1 significant latent variable (one predictive interclass) was based on 11 proteins. The 4 most important substances (i.e., VIP >1.0) are shown in Table 4. However, the model reliability was poor, since the CV-ANOVA did not reach significance.

**Table 4:**
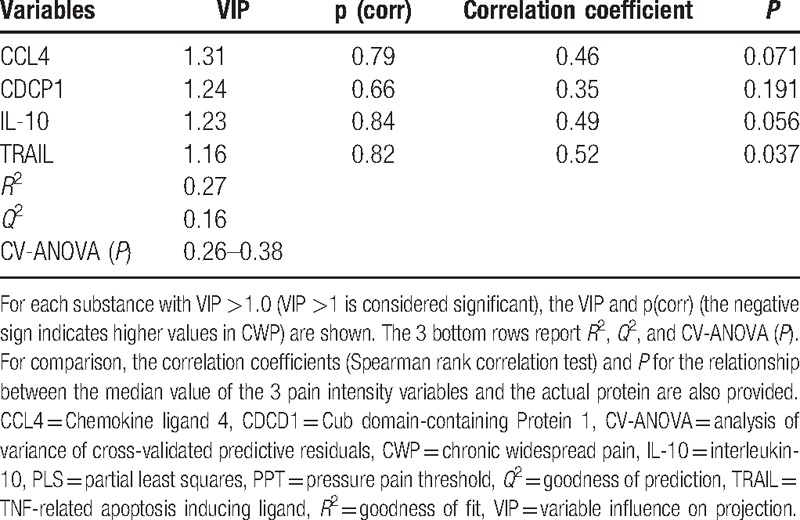
Simultaneous PLS regressions of pain intensity in the neck, shoulders, and low back (i.e., the 3 *y*-variables) in CWP using 11 proteins as regressors (*x*-variables).

Using only the 4 proteins shown in Table 4 as regressors, increased both *R*^2^ and *Q*^2^ (0.30 and 0.23, respectively) somewhat, but CV-ANOVA was still not significant (*P* values: 0.15–0.24).

## 5 Discussion

### 5.1 Major results

This explorative novel study found a pattern of inflammatory substances in the blood that clearly differentiated between CWP and CON, supporting that idea that CWP is associated with low-grade inflammation. Given the prevalence of CWP in the population, understanding its pathophysiological mechanisms is arguably a high priority. The present study contributes to this knowledge. A review recently pointed out the lack of reliable diagnostic or prognostic biomarkers for FMS.^[45]^ Using a panel enabling the measurement of 92 inflammation-related proteins at the same time, we have established the plasma inflammatory profile of CWP patients compared to healthy controls.

### 5.2 Proteins important for group separation

This study identified proteins deemed suitable for exploratory studies for the discovery of novel inflammation-related biomarkers (http://www.olink.com/proseek-multiplex/inflammation/; accessed July 6, 2016). Although the significant proteins in Table 2 are absent from the list prepared by Rodriguez-Pinto et al,^[25]^ some of these proteins are described in the broader pain and inflammatory literature. The “Supplemental Digital Content 2” briefly describes the different important proteins (i.e., those with a VIP >1.0; Table 2) for the group separation (CON or CWP) in relation to pain, nociception, and inflammation. We identified several important biomarkers: 3 chemokines (macrophage inflammatory protein [MIP1-α], mucosa-associated epithelial chemokine [CCL28], and CCL20), 1 cytokine (Cub domain-containing protein 1 [CDCP1]), 2 growth factors (LAP TGF-1 and hepatocyte growth factor [HGF]), as well as proteins involved in control of protein translation and sensitivity of neurons (4E-BP1), regulation of endosomal-lysosomal degradation pathway (STAMPB), Wnt signaling transduction pathways (AXIN1), caspase (Caspase-8), and regulation of NF-kappaB (sirtuin 2 [SIRT2]). Interestingly, there was a high degree of overlap compared to the results reported by Bäckryd et al in a cohort of FMS patients^[46]^; their top 4 proteins were STAM-binding protein (STAMBP), SIRT2, CD40, and AXIN1. Three of these proteins were also found in the present study (Table 2). Hence, in 2 different cohorts of FMS/CWP patients compared to 2 different healthy control groups, roughly the same inflammatory profile has been described in plasma. It is noteworthy that all the significant proteins in Table 2, except CCL28, also were significantly increased in patients with high pain intensity 1 year after disc herniation compared with those without or with very low pain intensity.^[28]^ This finding suggests that some of the reported alterations are common across pain conditions, whereas others may be condition-specific.

Several reports indicate that chronic pain conditions may be associated with chronic low-grade inflammation as revealed in measurements of, for example, cytokines in blood.^[47–49]^ Cytokines can be divided into proinflammatory and anti-inflammatory cytokines, and many different cytokines have been investigated in FMS (Rodiguez-Pinto et al).^[25]^ Increased serum levels of IL-6 and IL-8 have been reported in several studies of FMS.^[50]^ A recent study has reported that in addition to elevated IL-6 and tumor necrosis factor (TNF), elevated levels of the neuropeptides cortictropin-releasing hormone, substance P (SP), and SP-structurally related hemokin-1 have been found in serum.^[51]^ Impaired cell-mediated immunity (peripheral blood mononuclear cells) has been reported in FMS.^[52]^ However, some studies, including this study (see table in Supplemental Digital Content 1), report no differences in these cytokine concentrations in blood.^[52–54]^ Hence, in the present study, it was not possible to confirm significant elevations of IL-6 or IL-8. It must be pointed out that there is a certain degree of complexity concerning the interpretation of IL-6 alterations. Muscles are the main source of this cytokine in plasma.^[55]^ During exercise, there is a sharp (up to 100-fold) but short increase in plasma concentrations of IL-6, which is not related to muscle damage.^[56,57]^ Importantly, although exercise itself leads to an acute increase in IL-6 production and release, exercise training leads to reduced baseline circulating IL-6 levels.^[56]^ Hence, the interpretation of IL-6 levels in blood may be complex, requiring highly standardized mapping of activity levels. Although cytokines may have a role in the pathophysiology of FMS/CWP,^[25]^ statistical association obviously does not equate to causation; moreover, few longitudinal studies are available in this field.^[58]^ It is also notable that none of the proteins in Table 2 of the present study are present in the list of the previously studied cytokines presented by Rodriguez-Pintó et al.^[25]^ If our results are not all false-positives, it can be concluded that there is value to the strength of a broad hypothesis-generating approach to biomarker studies as opposed to a narrow hypothesis-driven analysis of a more limited number of substances. Some of the proteins in the table in “Supplemental Digital Content 1”—that is, fibroblast growth factor 23 , IL-10 receptor subunit beta, transforming growth factor alpha (TGF-alpha), and TNF ligand superfamily member 14—were associated with significant group differences; however, in the multivariate context, they could not explain group belonging. Traditional statistical methods may have limited value when analyzing several inflammatory markers,^[25]^ as the solutions to these problems (e.g., Bonferroni correction) do not consider that the investigated substances may be multivariately interdependent and intercorrelated.^[30]^ Using the false discovery rate may be associated with considerable loss of power and identification of true-positives.^[30]^ MVDA, which is used in the present study, takes the internal correlation structure of the data into consideration and considerably mitigates but does not completely eliminate the problem of multiple testing.^[31]^

### 5.3 Pain thresholds

As is obvious from Table 1, marked differences existed in the 4 PPT variables. Hence, the semiobjective variable PPT makes it possible to easily assign a subject to either of the 2 groups of subjects (i.e., CWP or CON). It was expected and found that the regressors (i.e., the proteins) of PPTs in all subjects taken together (Table 3) should largely be the same as in the regression of group membership (Table 2). However, it was not possible to regress the within-group variability of PPTs using the proteins as regressors.

### 5.4 Regression of pain intensities

It was possible to regress pain intensity, and the important proteins in Table 4 showed positive correlations with pain intensities. Hence, chemokine (CCL4), cytokines (IL-10 and CDCP1), and a protein of the TNF superfamily (TRAIL) correlated with pain intensities. These are briefly discussed in “Supplemental Digital Content 3.” Only CDCP1 (second important in Table 4) was also among the important proteins in the regression of group membership. Although these proteins have been described in the literature, they are generally not evaluated in correlation analyses vis-a-vis, for example, pain intensity.

## 6 Limitations

The results of this study need to be confirmed using other methods such as enzyme-linked immunosorbent assay and/or LUMINEX and through larger studies covering a broader spectrum of severity of CWP/FMS. The present cohort of CWP/FMS had a relatively low negative impact with respect to psychological factors (Table 1). Generally, CWP/FMS is associated with increased prevalence of psychological comorbidities, but the fact that this study is part of a comprehensive project may have led to selection of patients with relatively few negative implications. Future studies using this inflammatory panel should include patients with different degrees of pain spreading on the body. Future and necessarily larger studies should analyze the extent that chronic pain shares inflammation as a common mediator with common comorbidities. Several reports link certain conditions to low-grade inflammation. For example, chronic inflammation may be copresent with other chronic pain conditions, obesity, metabolic syndrome, and mental health disorders (e.g., depression and anxiety); that is, these conditions in different constellations may have inflammatory activity in common.^[59–63]^ Hence, it will be important to consider the above co-morbidities and determine unique and common regressors.

As always, there are potential confounding factors to consider. Although a difference in mean/median values of age was evident, this difference did not reach statistical significance in the present study (Table 1). Because 15 of 63 cytokines in plasma were associated with age,^[64]^ studies should include age-matched controls. Of the proteins listed in Table 2, 3 have previously been shown to be positively associated with age:^[64]^ CDCP1 (rho = 0.783); HGF (rho = 0.475); and TGF-β1 (rho = 0.368). However, apart from CCL28 (which was not analyzed), none of the other proteins listed in Table 2 had any correlation with age (cf. Larsson et al).^[64]^ If overweight/obesity and CWP are both associated with inflammation, it can be questioned whether BMI should be considered a confounder. At any rate, peripheral levels of cytokines have been associated with BMI, and in the present study, median BMI differed by a value of 3.9 (Table 1), which may appear substantial, but it did not reach statistical significance.^[65]^ Most importantly, for the proteins listed in Table 2, Larsson et al found that Spearman correlation coefficients (rho) ranged only between 0.003 and 0.253, making a large-scale confounding effect of BMI in the present study not especially likely (the range of BMI in the Larsson et al's study was 16.8–42.2). Although it is possible that age and BMI exerted some minor influence on our findings, we do not think it is likely that they influenced the results in a major way.

### 6.1 Perspective

Conceptually, there are at least 3 ways to biochemically study the mechanisms of FMS/CWP: locally via muscle biopsies or microdialysis in muscle tissue^[18,19]^; systemically using blood samples as in the present study; and via central nervous system, events as reflected in cerebrospinal fluid.^[66]^ All in all, a broad picture emerges that characterizes FMS/CWP according to inflammation processes in muscle tissue, systemic low-grade inflammation, and central neuroinflammation. Biomarker studies of this kind are arguably an important complement to psychophysical and imaging studies that endeavor to better understand the pathophysiology of FMS/CWP and contribute to substantiating the biological part of the biopsychosocial model of chronic pain. A better understanding of these (neuro)biological alterations may be a prerequisite for improving outcomes of long-term treatments/interventions of chronic pain conditions such as CWP/FMS. The pharmacological treatments of CWP/FMS often are clinically delivered using a trial-and-error concept. At best, small- to medium-sized effect sizes are found for nonpharmacological interventions.

## 7 Conclusions

This explorative study found a pattern of inflammatory substances in the blood that clearly differentiated patients with CWP from their healthy controls. Moreover, certain proteins also correlated with pain intensity in CWP, although no significant correlations existed with pain thresholds in CWP. The results of this study support the suggestion that CWP is associated with low-grade systemic inflammation. Future larger studies are needed to confirm the results and to investigate which alterations are condition-specific and which are common across chronic pain conditions. As it has been suggested that prevalent comorbidities to pain (e.g., depression, anxiety, poor sleep, tiredness) also are associated with inflammation, it will be important to determine whether inflammation is a common mediator.

## Supplementary Material

Supplemental Digital Content
