# Supplementary material for: Signs of ongoing inflammation in female patients with chronic widespread pain: A multivariate, explorative, cross-sectional study of blood samples
Source: Medicine (Baltimore). 2017 Mar 3;96(9):e6130. doi: 10.1097/MD.0000000000006130 (PMC5340439; doi:10.1097/MD.0000000000006130)
Supplement: Supplemental Digital Content [file medi-96-e6130-s001.doc]

# **Supplemental Digital Content 1**

Supplemental Digital Content 1: The table shows the panel of 92 proteins.

Table

Median values for the proteins (n=92) of the multiplex proximity extension assay (PEA) panel for CON and CWP. The ratio between the median values is also presented together with the statistical comparison (Mann-Whitney U test) between the two groups (p-value). * denotes significant difference; md = missing data. NA = not applicable.

| ***Protein*** | ***CON***  ***(Median)*** | ***CWP (Median)*** | ***CWP/CON***  ***ratio*** | ***Statistics***  ***p-value*** |
| --- | --- | --- | --- | --- |
| Adenosine Deaminase (ADA) | 10.56 | 11.61 | 1.10 | 0.236 |
| Artemin (ARTN) | md | md | NA | NA |
| Axin-1 (AXIN1) | 1.34 | 1.84 | 1.38 | >0.001* |
| Beta-nerve growth factor (Beta-NGF) | 0.85 | 0.88 | 1.04 | 0.058 |
| Brain-derived neurotrophic factor (BDNF) | 28.59 | 160.52 | 5.61 | 0.440 |
| Caspase 8 (CASP-8 ) | 0.76 | 0.89 | 1.18 | 0.005* |
| C-C motif chemokine 19 (CCL19) | 208.56 | 318.52 | 1.53 | 0.276 |
| C-C motif chemokine 20 (CCL20) | 7.55 | 26.36 | 3.49 | 0.012* |
| C-C motif chemokine 23 (CCL23) | 289.31 | 351.36 | 1.21 | 0.236 |
| C-C motif chemokine 25 (CCL25) | 23.56 | 22.11 | 0.94 | 0.718 |
| C-C motif chemokine 28 (CCL28) | 0.90 | 1.00 | 1.11 | 0.042* |
| C-C motif chemokine 4 (CCL4 ) | 13.13 | 14.97 | 1.14 | 0.149 |
| CD40L receptor (CD40) | 165.47 | 192.08 | 1.16 | 0.077 |
| CUB domain-containing protein 1 (CDCP1) | 1.70 | 2.12 | 1.24 | 0.030* |
| C-X-C motif chemokine 1 (CXCL1) | 123.11 | 65.61 | 0.53 | 0.124 |
| C-X-C motif chemokine 10 (CXCL10) | 102.79 | 100.86 | 0.98 | 0.765 |
| C-X-C motif chemokine 11 (CXCL11) | 44.47 | 44.22 | 0.99 | 0.863 |
| C-X-C motif chemokine 5 (CXCL5) | 534.21 | 502.34 | 0.94 | 0.789 |
| C-X-C motif chemokine 6 (CXCL6) | 41.07 | 38.49 | 0.94 | 0.937 |
| C-X-C motif chemokine 9 (CXCL9 ) | 44.89 | 40.32 | 0.90 | 0.912 |
| Cystatin D (CST5) | 39.08 | 35.09 | 0.90 | 0.539 |
| Delta and Notch-like epidermal growth factor-related recep (DNER) | 65.55 | 67.50 | 1.03 | 0.648 |
| Eotaxin-1 (CCL11) | 53.52 | 74.10 | 1.38 | 0.440 |
| Eukaryotic translation initiation factor 4E-binding protein 1 (4E-BP1) | 9.44 | 16.49 | 1.75 | 0.011* |
| Fibroblast growth factor 19 (FGF-19) | 127.52 | 152.94 | 1.20 | 0.305 |
| Fibroblast growth factor 21 (FGF-21) | 3.22 | 6.47 | 2.01 | 0.095 |
| Fibroblast growth factor 23 (FGF-23) | 1.99 | 2.37 | 1.19 | 0.021* |
| Fibroblast growth factor 5 (FGF-5) | 1.21 | 1.35 | 1.12 | 0.003* |
| Fms-related tyrosine kinase 3 ligand (Flt3L) | 173.74 | 170.59 | 0.98 | 0.888 |
| Fractalkine (CX3CL1) | 24.49 | 19.81 | 0.81 | 0.140 |
| Glial cell line-derived neurotrophic factor (hGDNF) | 1.98 | 2.07 | 1.05 | 0.863 |
| Hepatocyte growth factor (HGF) | 27.98 | 32.66 | 1.17 | 0.049* |
| Interferon gamma (IFN-gamma) | 0.91 | 0.90 | 0.99 | 0.888 |
| Interleukin-1 alpha (IL-1 alpha) | md | md | NA | NA |
| Interleukin-10 (IL-10) | 2.97 | 2.90 | 0.98 | 1.000 |
| Interleukin-10 receptor subunit alpha (IL-10RA) | md | md | NA | NA |
| Interleukin-10 receptor subunit beta (IL-10RB) | 22.18 | 17.96 | 0.81 | 0.042* |
| Interleukin-12 subunit beta (IL-12B) | 6.75 | 7.74 | 1.15 | 0.648 |
| Interleukin-13 (IL-13) | md | md | NA | NA |
| Interleukin-15 receptor subunit alpha (IL-15RA) | 0.88 | 0.88 | 1.01 | 0.369 |
| Interleukin-17A (IL-17A) | 0.43 | 0.44 | 1.00 | 0.888 |
| Interleukin-17C (IL-17C) | 1.38 | 1.45 | 1.05 | 0.987 |
| Interleukin-18 (IL-18) | 79.58 | 93.32 | 1.17 | 0.189 |
| Interleukin-18 receptor 1 (IL-18R1) | 34.97 | 38.37 | 1.10 | 0.440 |
| Interleukin-2 (IL-2) | md | md | NA | NA |
| Interleukin-2 receptor subunit beta (IL-2RB) | md | md | NA | NA |
| Interleukin-20 (IL-20) | md | md | NA | NA |
| Interleukin-20 receptor subunit alpha (IL-20RA) | md | md | NA | NA |
| Interleukin-22 receptor subunit alpha-1 (IL-22 RA1) | md | md | NA | NA |
| Interleukin-24 (IL-24) | md | md | NA | NA |
| Interleukin-33 (IL-33) | md | md | NA | NA |
| Interleukin-4 (IL-4) | md | md | NA | NA |
| Interleukin-5 (IL-5) | md | md | NA | NA |
| Interleukin-6 (IL-6) | 1.56 | 2.13 | 1.37 | 0.168 |
| Interleukin-7 (IL-7) | 1.99 | 1.96 | 0.99 | 0.741 |
| Interleukin-8 (IL-8) | 19.33 | 17.74 | 0.92 | 0.912 |
| Latency-associated peptide transforming growth factor beta 1 (LAP TGF-beta-1) | 24.35 | 27.68 | 1.14 | 0.026* |
| Leukaemia inhibitory factor (LIF) | md | md | NA | NA |
| Leukaemia inhibitory factor receptor (LIF-R) | 4.03 | 3.19 | 0.79 | 0.046* |
| Macrophage colony-stimulating factor 1 (CSF-1) | 88.37 | 89.00 | 1.01 | 0.838 |
| Macrophage inflammatory protein 1-alpha (MIP-1 alpha) | 1.27 | 1.77 | 1.39 | 0.003* |
| Matrix metalloproteinase-1 (MMP-1) | 1.29 | 1.57 | 1.22 | 0.236 |
| Matrix metalloproteinase-10 (MMP-10) | 34.44 | 39.51 | 1.15 | 0.336 |
| Monocyte chemotactic protein 1 (MCP-1) | 330.14 | 354.37 | 1.07 | 0.320 |
| Monocyte chemotactic protein 2 (MCP-2) | 122.44 | 113.55 | 0.93 | 0.838 |
| Monocyte chemotactic protein 3 (MCP-3) | 1.12 | 1.21 | 1.07 | 0.320 |
| Monocyte chemotactic protein 4 (MCP-4) | 1.13 | 1.37 | 1.21 | 0.023* |
| Natural killer cell receptor 2B4 (CD244) | 27.43 | 29.52 | 1.08 | 0.352 |
| Neurotrophin-3 (NT-3) | 1.66 | 1.60 | 0.96 | 0.498 |
| Neurturin (NRTN) | md | md | NA | NA |
| Oncostatin-M (OSM) | 3.27 | 3.57 | 1.09 | 0.519 |
| Osteoprotegerin (OPG) | 408.35 | 408.60 | 1.00 | 0.648 |
| Programmed cell death 1 ligand 1 (PD-L1) | md | md | NA | NA |
| Protein S100-A12 (EN-RAGE) | 0.92 | 0.81 | 0.89 | 0.539 |
| Signalling lymphocytic activation molecule (SLAMF1) | 2.09 | 2.18 | 1.04 | 0.789 |
| SIR2-like protein 2 (SIRT2) | 2.94 | 3.99 | 1.36 | 0.006* |
| STAM-binding protein (STAMPB) | 4.07 | 5.46 | 1.34 | 0.005* |
| Stem cell factor (SCF) | 118.52 | 120.22 | 1.01 | 0.626 |
| Sulfotransferase 1A1 (ST1A1) | md | md | NA | NA |
| T cell surface glycoprotein CD6 isoform (CD6) | 6.94 | 7.89 | 1.14 | 0.236 |
| T-cell surface glycoprotein CD5 (CD5) | 4.42 | 4.64 | 1.05 | 0.765 |
| Thymic stromal lymphopoietin (TSLP) | md | md | NA | NA |
| TNF-beta (TNFB) | 5.09 | 4.75 | 0.93 | 0.671 |
| TNF-related activation-induced cytokine (TRANCE) | 7.03 | 7.52 | 1.07 | 0.814 |
| TNF-related apoptosis-inducing ligand (TRAIL) | 150.36 | 164.25 | 1.09 | 0.626 |
| Transforming growth factor alpha (TGF-alpha) | 0.76 | 0.97 | 1.28 | <0.001* |
| Tumour necrosis factor (Ligand) superfamily, member 12 (TWEAK) | 199.28 | 208.61 | 1.05 | 0.178 |
| Tumour necrosis factor (TNF) | md | md | NA | NA |
| Tumour necrosis factor ligand superfamily member 14 (TNFSF14) | 1.24 | 1.61 | 1.30 | 0.023* |
| Tumour necrosis factor receptor superfamily member 9 (TNFRSF9) | 25.88 | 28.71 | 1.11 | 0.741 |
| Urokinase-type plasminogen activator (uPA) | 539.55 | 595.09 | 1.10 | 0.459 |
| Vascular endothelial growth factor A (VEGF-A) | 804.17 | 969.65 | 1.21 | 0.102 |

# **Supplemental Digital Content 2**

Supplemental Digital Content 2: The different proteins important for the group separation (CON or CWP) are briefly discussed in relation to pain, nociception, and inflammation.

This text briefly discusses the most important proteins for separating the two groups (CON or CWP) (**Table 2**) in relation to pain, nociception, and inflammation.

**MIP1-α (macrophage inflammatory protein-1α)**

This chemokine – also known as CCL3 – acts mainly through receptors CCR1 and CCR5 ^1^. This chemokine is upregulated in rat and human UVB models of inflammatory pain in the skin ^2^. Subjects with chronic pelvic syndrome had increased levels of MIP1-α in prostatic secretions ^3^. Peripheral and central nervous system injuries cause up-regulation of MIP1-α and CCR1 in the spinal cord and the level remains elevated for several weeks ^4, 5^. MIP1-α can modulate pain sensitivity ^1^, and intraplantar injection of MIP1-α results in pain-related hypersensitivity. Subjects with work-related neck-shoulder pain had no significant increase in serum MIP1-α, but the MIP1-α level correlated with pain intensity ^6^. Moreover, no difference in plasma MIP1-α was found in a study of patients with FMS ^7^, but these patients had decreased peripheral blood mononuclear cell responses in MIP1-alpha and other cytokines.

**CCL28 (Mucosa-associated epithelial chemokine)**

CCL28 was originally identified as a product of epithelial cells and binds the receptor CCR10 ^8^. CCL28 participates in leukocyte-mediated inflammation, homeostasis, and regulation of cellular movements ^9^ and has antimicrobial activity. Pro-inflammatory cytokines (e.g., IL-1β, IL-6, and TNF-α) and bacterial products increase the levels of CCL28 ^9, 10^. Several studies indicate that CCL28 levels are higher in samples from patients with inflammatory diseases including RA and OA synovial tissue ^9, 11, 12^.

**4E-BP1 (Eukaryotic initiation factor 4E-binding protein 1)**

mTOR kinase is a conserved serine/threonine protein kinase belonging to the phosphoinositide 3-kinase family (PI3K) that regulates multiple processes in different tissues ^13, 14^. It forms two distinct complexes, mTORC1 and mTORC2 ^14^. Activation of mTORC1 is associated with translation initiation and subsequent protein synthesis ^14^. mTORC1 is activated by different factors, e.g., cytokines, growth factors, energy status to control functions associated with cell growth, and metabolism. The down-stream 4E-BP1 protein of mTORC1 together with S6 kinase 1 (S6K1) are important components in the protein translation processes ^13, 15^. That mTORC1 and its downstream components (4E-BP1) can control protein translation is associated with an ability to regulate the sensitivity of peripheral and central sensory neurons ^13, 16^. Peripheral inflammation increases mTOR activity in the dorsal horn and it has been suggested and partly confirmed that inhibition of mTOR activity (and thereby downstream substances) may have beneficial effects with respect to maintenance, mechanical hypersensitivity, and/or opioid-induced tolerance in chronic pain conditions ^13, 16, 17^.

**STAMBP (STAM-binding protein)**

STAMBP also known as AMSH (associated molecule with Src homology 3 domain of STAM) is zinc metalloprotease. Ubiquitination – attachment of ubiquitin to proteins – controls several cellular functions such as protein quality control, cell cycle progression, transcription, endocytosis, DNA repair, and cellular signalling ^18^. STAMBP is a deubiquitinating enzyme, which can remove ubiquitin from proteins ^18^. Endocytic sorting of cell-surface receptors to lysosomes for their degradation is executed by ESCRT (endosomal sorting complexes required for transport) machinery ^18^. STAMBP is recruited to ESCRT to regulate the endosomal-lysosomal degradation pathway ^19, 20^. Mutations in STAMBP gene have been linked to the neural loss underlying microcephalycapillary malformation syndrome ^21^.

**AXIN 1**

Axin 1 is involved in down-regulation of Wnt signalling transduction pathways, which pass signals into a cell through surface receptors. These pathways regulate gene transcription, cytoskeleton, and calcium inside the cell. Wnts can activate the intracellular β-canetin-dependent pathway, which involves β-canetin-mediated gene regulation ^22^. There are also β-canetin-independent pathways that act via cell surface G protein-coupled receptors (Frizzleds); for references: ^22^. The Wnt family of proteins are involved in the regular processes during nervous system development and has an important role in the pathogenesis of neuropathic pain ^23-25^. Wnt signalling regulates different functions in the immune system, e.g., peripheral immune cells; for references: ^26^. Activated Wnt signalling stimulates production of pro-inflammatory cytokines IL-18 and TNF-α ^23^. Axin is a negative regulator of the Wnt/β-canetin-pathway and together with Glycogen Synthase Kinase (GSK) and adenomatous polyposis coli (APC) a complex is formed that destroys β-Canetin and ultimately restrains transcription. Spinal blockade of Wnt signalling resulted in inhibited production and persistence of neuropathic pain in rodents ^23^. It has been suggested that the Wnt/β-canetin-dependent pathway participates in the pathogenesis of several CNS disorders (cerebral ischemia, psychiatric disorders, Alzheimer’s disease. and epilepsy) ^24, 27^. Axin 1 has been reported to inhibit Salmonella invasion and bacterial inflammation ^28^. Recent studies indicate that axin 1 is also involved in other signalling pathways ^28^.

**LAP TGF-beta1**

Transforming growth factor β1 (TGF-β1) is a polypeptide and a member of the transforming growth factor beta superfamily of cytokines ^29^. Three directions of its activities have been identified: 1) cell proliferation, growth, differentiation and cell movement; 2) immunomodulatory effects; and 3) profibrogenic effects ^30^. The actions of TGF-β1 can be local and systemic ^30^. TGF-β1 has protective effects against the development of chronic neuropathic pain by inhibiting neuroimmune responses of neurons and glia cells and promoting the expression of endogenous opioids within the spinal cord ^31^. There are also reports that TGF-β1 has peripheral anti-nociceptive effects and participate in the prevention of peripheral anti-nociceptive hypersensitization following nerve injury ^29^.

**HGF (Hepatocyte growth factor**)

HGF is a secreted by mesenchymal cells of the liver, lung, central nervous system, and the intestine ^32^. It promotes endothelial cell proliferation and angiogenesis to induce collateral formation in preclinical models of peripheral arterial disease ^33^. HGF also has anti-inflammatory effects; for references: ^32, 34, 35^. Intramuscular injection of naked plasmid DNA (pUDK-HGF) expressing human hepatocyte growth factor (HGF) in patients with critical limb ischemia significantly reduced pain ^33^. Intramuscular injections of plasmid DNA expressing two isoforms of HGF in subjects with painful diabetic peripheral neuropathy was associated with significant pain reductions at follow-ups several months later ^36^. In rat models, HGF reduced IBD symptomatology, diminished intestinal injury scores in IBD, and reduced number of days with pain in induced colitis; for references: ^32^. Patients with disc herniation had significantly lower HGF plasma levels than a group with other diagnoses including spinal stenosis and degenerative disc disease ^34^. The latter group exhibited improvement in pain intensity that was correlated with change in HGF level.

**CDCP1 (Cub Domain-containing Protein 1)**

The cytokine CDCP1 – also known as SIMA135 or CD318 – is a type-I transmembrane glycoporotein. Plasma level of CDCP1 increases with age ^37^. It is highly expressed in various human cancer cells ^38, 39^ and is considered a master regulator of the metastatic and invasive potential of solid cancers due to its control of anoikis resistance, cell migration, cell invasion, matrix metalloproteinase secretion, and invadopodia formation ^39^. The lack of knowledge concerning its mode of action, partners, and the signalling pathways downstream of CDCP1 have been raised ^38^. Hence, there are both suggestions that it acts as an oncogene or as a tumour suppressor ^38^. Our results indicate that CDCP1 is also involved in non-malignant inflammatory processes.

**CASP8 (Caspase-8)**

Caspases are a family of proteases that have been traditionally seen as either involved in apoptosis or in inflammation ^40^. Caspase-8 has an important role in inhibition of inflammatory cell death pathways (necroptosis)^40^. Inflammasome-induced caspase-1 activation is an important source of IL-1β, but caspase-8 has also been identified as a protease that can process IL-1β either in the inflammasomes or independently ^40, 41^. Hence, caspase-8 can engage multiple proteins depending both on stimuli and the actual cell types.

**CCL20 (chemokine (C-C motif) ligand 20)**

CCL20 – also known as macrophage inflammatory protein 3α (MIP-3α) – functions both as an inflammatory and homeostatic chemokine ^42^. Chemokine receptor 6 (CCR6) is the sole receptor of this chemokine ^42, 43^. CCL20 can be strongly induced by pro-inflammatory signals and is highly expressed in the synovial fluid of RA and in OA cartilage ^43, 44^. It can augment inflammation of RA ^43^. Bidirectional interactions may exist between CCL20 and IL-1β, the major cytokine implicated in the pathogenesis in OA ^44^.

**SIRT2 (Sirtuin 2)**

Sirtuins are a family of NAD+ dependent deacylases ^45^. Seven mammalian homologs have been identified (SIRT1-7). Out of these, SIRT2 is the only primarily cytoplasmic isoform, but it can also be found in the nucleus ^46^. It is expressed in several tissues and especially in metabolically relevant tissues and organs such as brain, muscles, and liver. Several studies of animal models of Parkinson´s disease, Huntington´s disease, and ischemia stroke have suggested potential neuroprotective effects of SIRT2 inhibition or deficiency (for references). SIRT2 can directly bind, deacetylate, and inhibit the p65 subunit of NF-kappaB (nuclear factor kappa-light-chain-enhancer of activated B cells), indicating an important role in inflammation. NF-kappaB is a major transcriptional regulator of the inflammatory response. SIRT2 may play a role in peripheral myelination ^47^. Wang et al. concluded that SIRT2 could produce contrasting roles in cell death and oxidative stress under different conditions and, like calcium, play complex roles in biological processes ^48^.

**References**

1. Dawes JM, McMahon SB. Chemokines as peripheral pain mediators. *Neurosci Lett*. 2013;557 Pt A:1-8.

2. Dawes JM, Calvo M, Perkins JR, et al. CXCL5 mediates UVB irradiation-induced pain. *Sci Transl Med*. 2011;3:90ra60.

3. Desireddi NV, Campbell PL, Stern JA, et al. Monocyte chemoattractant protein-1 and macrophage inflammatory protein-1alpha as possible biomarkers for the chronic pelvic pain syndrome. *J Urol*. 2008;179:1857-61; discussion 61-2.

4. Knerlich-Lukoschus F, von der Ropp-Brenner B, Lucius R, et al. Spatiotemporal CCR1, CCL3(MIP-1alpha), CXCR4, CXCL12(SDF-1alpha) expression patterns in a rat spinal cord injury model of posttraumatic neuropathic pain. *J Neurosurg Spine*. 2011;14:583-97.

5. Kiguchi N, Kobayashi Y, Maeda T, et al. CC-chemokine MIP-1alpha in the spinal cord contributes to nerve injury-induced neuropathic pain. *Neurosci Lett*. 2010;484:17-21.

6. Matute Wilander A, Karedal M, Axmon A, et al. Inflammatory biomarkers in serum in subjects with and without work related neck/shoulder complaints. *BMC Musculoskelet Disord*. 2014;15:103.

7. Behm FG, Gavin IM, Karpenko O, et al. Unique immunologic patterns in fibromyalgia. *BMC Clin Pathol*. 2012;12:25.

8. Wang W, Soto H, Oldham ER, et al. Identification of a novel chemokine (CCL28), which binds CCR10 (GPR2). *J Biol Chem*. 2000;275:22313-23.

9. Ertugrul AS, Sahin H, Dikilitas A, et al. Comparison of CCL28, interleukin-8, interleukin-1beta and tumor necrosis factor-alpha in subjects with gingivitis, chronic periodontitis and generalized aggressive periodontitis. *J Periodontal Res*. 2013;48:44-51.

10. Ogawa H, Iimura M, Eckmann L, et al. Regulated production of the chemokine CCL28 in human colon epithelium. *American journal of physiology Gastrointestinal and liver physiology*. 2004;287:G1062-9.

11. Zlotnik A, Yoshie O. The chemokine superfamily revisited. *Immunity*. 2012;36:705-16.

12. Chen Z, Kim SJ, Essani AB, et al. Characterising the expression and function of CCL28 and its corresponding receptor, CCR10, in RA pathogenesis. *Ann Rheum Dis*. 2015;74:1898-906.

13. Lisi L, Aceto P, Navarra P, et al. mTOR kinase: a possible pharmacological target in the management of chronic pain. *Biomed Res Int*. 2015;2015:394257.

14. Steiner JL, Lang CH. Alcohol impairs skeletal muscle protein synthesis and mTOR signaling in a time-dependent manner following electrically stimulated muscle contraction. *Journal of applied physiology (Bethesda, Md : 1985)*. 2014;117:1170-9.

15. Dent JR, Edge JA, Hawke E, et al. Sex differences in acute translational repressor 4E-BP1 activity and sprint performance in response to repeated-sprint exercise in team sport athletes. *J Sci Med Sport*. 2015;18:730-6.

16. Obara I, Tochiki KK, Geranton SM, et al. Systemic inhibition of the mammalian target of rapamycin (mTOR) pathway reduces neuropathic pain in mice. *Pain*. 2011;152:2582-95.

17. Lutz BM, Nia S, Xiong M, et al. mTOR, a new potential target for chronic pain and opioid-induced tolerance and hyperalgesia. *Mol Pain*. 2015;11:32.

18. Shrestha RK, Ronau JA, Davies CW, et al. Insights into the mechanism of deubiquitination by JAMM deubiquitinases from cocrystal structures of the enzyme with the substrate and product. *Biochemistry*. 2014;53:3199-217.

19. Clague MJ, Urbe S. Endocytosis: the DUB version. *Trends Cell Biol*. 2006;16:551-9.

20. Davies CW, Paul LN, Das C. Mechanism of recruitment and activation of the endosome-associated deubiquitinase AMSH. *Biochemistry*. 2013;52:7818-29.

21. McDonell LM, Mirzaa GM, Alcantara D, et al. Mutations in STAMBP, encoding a deubiquitinating enzyme, cause microcephaly-capillary malformation syndrome. *Nat Genet*. 2013;45:556-62.

22. Simonetti M, Agarwal N, Stosser S, et al. Wnt-Fzd signaling sensitizes peripheral sensory neurons via distinct noncanonical pathways. *Neuron*. 2014;83:104-21.

23. Zhang YK, Huang ZJ, Liu S, et al. WNT signaling underlies the pathogenesis of neuropathic pain in rodents. *J Clin Invest*. 2013;123:2268-86.

24. Itokazu T, Hayano Y, Takahashi R, et al. Involvement of Wnt/beta-catenin signaling in the development of neuropathic pain. *Neurosci Res*. 2014;79:34-40.

25. Liu S, Liu YP, Huang ZJ, et al. Wnt/Ryk signaling contributes to neuropathic pain by regulating sensory neuron excitability and spinal synaptic plasticity in rats. *Pain*. 2015;156:2572-84.

26. Marchetti B, Pluchino S. Wnt your brain be inflamed? Yes, it Wnt! *Trends Mol Med*. 2013;19:144-56.

27. Libro R, Bramanti P, Mazzon E. The role of the wnt canonical signaling in neurodegenerative diseases. *Life Sci*. 2016.

28. Zhang YG, Wu S, Xia Y, et al. Axin1 prevents Salmonella invasiveness and inflammatory response in intestinal epithelial cells. *PLoS One*. 2012;7:e34942.

29. Lantero A, Tramullas M, Diaz A, et al. Transforming growth factor-beta in normal nociceptive processing and pathological pain models. *Mol Neurobiol*. 2012;45:76-86.

30. Kajdaniuk D, Marek B, Borgiel-Marek H, et al. Transforming growth factor beta1 (TGFbeta1) in physiology and pathology. *Endokrynol Pol*. 2013;64:384-96.

31. Echeverry S, Shi XQ, Haw A, et al. Transforming growth factor-beta1 impairs neuropathic pain through pleiotropic effects. *Mol Pain*. 2009;5:16.

32. Zwintscher N, Shah P, Salgar S, et al. Hepatocyte growth factor, hepatocyte growth factor activator and arginine in a rat fulminant colitis model. *Ann Med Surg (Lond)*. 2016 7:97-103.

33. Cui S, Guo L, Li X, et al. Clinical Safety and Preliminary Efficacy of Plasmid pUDK-HGF Expressing Human Hepatocyte Growth Factor (HGF) in Patients with Critical Limb Ischemia. *Eur J Vasc Endovasc Surg*. 2015 50:494-501.

34. Weber KT, Satoh S, Alipui DO, et al. Exploratory study for identifying systemic biomarkers that correlate with pain response in patients with intervertebral disc disorders. *Immunol Res*. 2015;63:170-80.

35. Zhang J, Middleton K, Fu F, et al. HGF mediates the anti-inflammatory effects of PRP on injured tendons. *PLoS One*. 2013 8:e67303.

36. Ajroud-Driss S, Christiansen M, Allen J, et al. Phase 1/2 open-label dose-escalation study of plasmid DNA expressing two isoforms of hepatocyte growth factor in patients with painful diabetic peripheral neuropathy. *Mol Ther*. 2013;21:1279-86.

37. Larsson A, Carlsson L, Gordh T, et al. The effects of age and gender on plasma levels of 63 cytokines. *J Immunol Methods*. 2015;425:58-61.

38. Gandji LY, Proust R, Larue L, et al. The tyrosine phosphatase SHP2 associates with CUB domain-containing protein-1 (CDCP1), regulating its expression at the cell surface in a phosphorylation-dependent manner. *PLoS One*. 2015;10:e0123472.

39. Uekita T, Sakai R. Roles of CUB domain-containing protein 1 signaling in cancer invasion and metastasis. *Cancer Sci*. 2011;102:1943-8.

40. Creagh EM. Caspase crosstalk: integration of apoptotic and innate immune signalling pathways. *Trends Immunol*. 2014;35:631-40.

41. Gurung P, Kanneganti TD. Novel roles for caspase-8 in IL-1beta and inflammasome regulation. *Am J Pathol*. 2015;185:17-25.

42. Williams IR. CCR6 and CCL20: partners in intestinal immunity and lymphorganogenesis. *Ann N Y Acad Sci*. 2006;1072:52-61.

43. Tanida S, Yoshitomi H, Nishitani K, et al. CCL20 produced in the cytokine network of rheumatoid arthritis recruits CCR6+ mononuclear cells and enhances the production of IL-6. *Cytokine*. 2009;47:112-8.

44. Alaaeddine N, Antoniou J, Moussa M, et al. The chemokine CCL20 induces proinflammatory and matrix degradative responses in cartilage. *Inflamm Res*. 2015;64:721-31.

45. Gomes P, Outeiro TF, Cavadas C. Emerging Role of Sirtuin 2 in the Regulation of Mammalian Metabolism. *Trends Pharmacol Sci*. 2015;36:756-68.

46. Vaquero A, Scher MB, Lee DH, et al. SirT2 is a histone deacetylase with preference for histone H4 Lys 16 during mitosis. *Genes Dev*. 2006;20:1256-61.

47. Beirowski B, Gustin J, Armour SM, et al. Sir-two-homolog 2 (Sirt2) modulates peripheral myelination through polarity protein Par-3/atypical protein kinase C (aPKC) signaling. *Proc Natl Acad Sci U S A*. 2011;108:E952-61.

48. Wang B, Zhang Y, Cao W, et al. SIRT2 plays significant roles in lipopolysaccharides-induced neuroinflammation and brain injury in mice. *Neurochem Res*. 2016;41:2490-500.

Supplemental Digital Content 3: The proteins significantly correlated with pain intensity in CWP are briefly discussed in relation to pain, nociception, and inflammation.

# **Supplemental Digital Content 3**

This text discusses the proteins with highest correlations with pain intensity **(Table 4).**

**CCL4 (Chemokine ligand 4)**

CCL4 was the most important regressor of pain intensity. It is also known as Macrophage inflammatory protein or 1β (MIP-1β). When a peripheral nerve is injured, leptin is highly expressed and released, which in turn upregulates the expression of, e.g., CCL4 ^1^. Behm et al. found no significant difference in CCL4 plasma level between FMS patients and healthy controls ^2^. They reported that cytokine levels for CCL4 were significantly lower in FMS after stimulation of peripheral blood mononuclear cells ^2^. As in our study (**Table 4**), a positive significant correlation has been reported between CCL4 and pain severity in FMS ^3^.

**CDCP1 (Cub Domain-containing Protein 1)**

See Supplemental Digital Content 2.

**IL-10**

This interleukin is produced by activated T cells, B cells, macrophages, mast cells, and kerationocytes ^4^. IL-10 regulates the expression of substance P ^5^. The production is inhibited by autoregulation of IL-10 and by IL-4, IL-13, and IFNgamma. Significantly lower levels of IL-10 in the blood (serum or plasma) have been reported in CWP/FMS ^4^ although other studies have not found differences or increased levels in IL-10 compared to healthy controls ^2, 6-8^. A systematic review in 2011 could not conclude altered levels in plasma or serum for IL-10 in FMS ^9^; a similar conclusion was drawn in a more recent review ^5^. No significant correlations were found between mRNA for IL-10 and pain intensity ^4^. A negative correlation existed between perceived fatigue and the plasma levels of this interleukin in FMS ^3^. No significant correlations have been found between IL-10 serum levels and psychological aspects (catastrophising, anxiety, and depression) or fibromyalgia impact questionnaire in FMS ^8, 10^.

**TRAIL (TNF-related apoptosis inducing ligand)**

TRAIL is a member of the TNF superfamily. It has been investigated for its anticancer activity, but it is also involved in different processes of the innate and adaptive immune system ^11, 12^. It can trigger cellular apoptosis in malignant, virally infected, and over-activated immune cells and necroptosis ^13^. In intervertebral disks, TRAIL correlated with the degenerative state of the disk and may delay progression of disk degeneration ^14^. TRAIL has been identified as a potential biomarker of psoriatic arthritis; higher levels were reported in serum and the levels correlated with CRP. TRAIL has also been discussed as a potential biomarker in cardiovascular diseases. Serum levels are significantly decreased in patients with or predisposed for cardiovascular diseases ^13^. Studies of animals suggest that TRAIL protects against atherosclerosis ^13^. Moreover, a recent review found that TRAIL plays a role in inflammatory disorders of the brain and preliminary evidence suggests a potential role of a circulating biomarker for neurocognitive impairment and depression ^11^.

**References**

1. Liou JT, Lee CM, Day YJ. The immune aspect in neuropathic pain: role of chemokines. *Acta Anaesthesiol Taiwan*. 2013;51:127-32.

2. Behm FG, Gavin IM, Karpenko O, et al. Unique immunologic patterns in fibromyalgia. *BMC Clin Pathol*. 2012;12:25.

3. Menzies V, Lyon DE, Elswick RK, Jr., et al. Psychoneuroimmunological relationships in women with fibromyalgia. *Biol Res Nurs*. 2013;15:219-25.

4. Uceyler N, Valenza R, Stock M, et al. Reduced levels of antiinflammatory cytokines in patients with chronic widespread pain. *Arthritis Rheum*. 2006;54:2656-64.

5. Rodriguez-Pinto I, Agmon-Levin N, Howard A, et al. Fibromyalgia and cytokines. *Immunology letters*. 2014;161:200-3.

6. Wang H, Moser M, Schiltenwolf M, et al. Circulating cytokine levels compared to pain in patients with fibromyalgia -- a prospective longitudinal study over 6 months. *J Rheumatol*. 2008;35:1366-70.

7. Bazzichi L, Rossi A, Massimetti G, et al. Cytokine patterns in fibromyalgia and their correlation with clinical manifestations. *Clin Exp Rheumatol*. 2007;25:225-30.

8. Ranzolin A, Duarte AL, Bredemeier M, et al. Evaluation of cytokines, oxidative stress markers and brain-derived neurotrophic factor in patients with fibromyalgia - A controlled cross-sectional study. *Cytokine*. 2016;84:25-8.

9. Uceyler N, Hauser W, Sommer C. Systematic review with meta-analysis: cytokines in fibromyalgia syndrome. *BMC Musculoskelet Disord*. 2011;12:245.

10. Sturgeon JA, Darnall BD, Zwickey HL, et al. Proinflammatory cytokines and DHEA-S in women with fibromyalgia: impact of psychological distress and menopausal status. *J Pain Res*. 2014;7:707-16.

11. Tisato V, Gonelli A, Voltan R, et al. Clinical perspectives of TRAIL: insights into central nervous system disorders. *Cell Mol Life Sci*. 2016;73:2017-27.

12. Cheng W, Zhao Y, Wang S, et al. Tumor necrosis factor-related apoptosis-inducing ligand in vascular inflammation and atherosclerosis: a protector or culprit? *Vascul Pharmacol*. 2014;63:135-44.

13. Bernardi S, Bossi F, Toffoli B, et al. Roles and Clinical Applications of OPG and TRAIL as Biomarkers in Cardiovascular Disease. *Biomed Res Int*. 2016;2016:1752854.

14. Bertram H, Nerlich A, Omlor G, et al. Expression of TRAIL and the death receptors DR4 and DR5 correlates with progression of degeneration in human intervertebral disks. *Mod Pathol*. 2009;22:895-905.
